# The BDNF/TrkB Signaling Pathway Is Involved in Heat Hyperalgesia Mediated by Cdk5 in Rats

**DOI:** 10.1371/journal.pone.0085536

**Published:** 2014-01-21

**Authors:** Hong-Hai Zhang, Xiao-Qin Zhang, Qing-Sheng Xue, Yan-Luo, Jin-Lu Huang, Su Zhang, Hai-Jun Shao, Han Lu, Wen-Yuan Wang, Bu-Wei Yu

**Affiliations:** 1 Department of Anesthesiology, Ruijin Hospital, Shanghai JiaoTong University School of Medicine, Shanghai, P. R. China; 2 Department of Anesthesiology, Hang Zhou First People's Hospital, Nan Jing Medical University, Zhejiang, P. R. China; 3 Department of Pharmacy, Sixth Affiliated Hospital of Shanghai JiaoTong University School of Medicine, Shanghai, P. R. China; University of Arizona, United States of America

## Abstract

**Background:**

Cyclin-dependent kinase 5 (Cdk5) has been shown to play an important role in mediating inflammation-induced heat hyperalgesia. However, the underlying mechanism remains unclear. The aim of this study was to determine whether roscovitine, an inhibitor of Cdk5, could reverse the heat hyperalgesia induced by peripheral injection of complete Freund's adjuvant (CFA) via the brain-derived neurotrophic factor (BDNF)-tyrosine kinase B (TrkB) signaling pathway in the dorsal horn of the spinal cord in rats.

**Results:**

Heat hyperalgesia induced by peripheral injection of CFA was significantly reversed by roscovitine, TrkB-IgG, and the TrkB inhibitor K252a, respectively. Furthermore, BDNF was significantly increased from 0.5 h to 24 h after CFA injection in the spinal cord dorsal horn. Intrathecal adminstration of the Cdk5 inhibitor roscovitine had no obvious effects on BDNF levels. Increased TrkB protein level was significantly reversed by roscovitine between 0.5 h and 6 h after CFA injection. Cdk5 and TrkB co-immunoprecipitation results suggested Cdk5 mediates the heat hyperalgesia induced by CFA injection by binding with TrkB, and the binding between Cdk5 and TrkB was markedly blocked by intrathecal adminstration of roscovitine.

**Conclusion:**

Our data suggested that the BDNF-TrkB signaling pathway was involved in CFA-induced heat hyperalgesia mediated by Cdk5. Roscovitine reversed the heat hyperalgesia induced by peripheral injection of CFA by blocking BDNF/TrkB signaling pathway, suggesting that severing the close crosstalk between Cdk5 and the BDNF/TrkB signaling cascade may present a potential target for anti-inflammatory pain.

## Introduction

Cyclin-dependent kinase 5 (Cdk5), one of the cyclin-dependent kinases (Cdks), plays a key role in heat hyperalgesia induced by inflammation [1]–[8]. Cdk5 was shown to mediate carrageenan-induced heat hyperalgesia by phosphorylating vanilloid receptor 1 (VR1) of dorsal root ganglion (DRG) in mice [2], [3]. Recent evidence demonstrated that Cdk5 was involved in CFA-induced heat hyperalgesia by controlling TRPV1 (transient receptor potential channel) membrane trafficking in rats [4]. We previously found that increased synaptophysin protein, an important presynaptic vesicle membrane protein that functions in the release of neurotransmitters, was involved in CFA-induced heat hyperalgesia mediated by Cdk5 in rats, suggesting that Cdk5 may control heat hyperalgesia induced by inflammation via regulating the release of neurotransmitters [7]. However, the mechanism by which Cdk5 mediates inflammation-induced heat hyperalgesia has not been explored in detail [8].

A growing body of evidence has indicated that the BDNF-TrkB signaling pathway plays a critical role in heat hyperalgesia of inflammatory pain [9], [10]. BDNF is synthesized in the primary sensory neurons and transported to the central terminals of the primary afferents in the spinal dorsal horn, where it is involved in modulating pain sensitization caused by different pain stimuli [11]. BDNF synthesis is significantly increased in different populations of DRG neurons during inflammatory and neuropathic pain, and TrkB protein levels were also remarkably increased during these processes [12], [13]. Furthermore, spinal intrathecal administration of BDNF-scavenging protein TrkB-IgG or K252a significantly attenuates nociceptive behavioral responses induced by inflammation [13], [14]. However, increased TrkB protein level in the spinal dorsal horn only persisted for several days post-inflammation treatment, indicating that the BDNF-TrkB pathway may function during the early phase of inflammatory pain [15].

Recent studies demonstrated that Cdk5 and its activator p35 is involved in the development of hippocampal neurons of mice by close interactions with TrkB [16]. Namely, in vitro administration of roscovitine significantly abolished the increase in surface TrkB protein level induced by glycine stimulation. TrkB surface recruitment was remarkably abrogated in hippocampal neurons in Cdk5 or p35 knockdown mice. Another study showed that activated Cdk5 increased the percentage of TrkB on the surface of hippocampal neurons in mice [17]. Given the accumulating evidence, we hypothesize that the BDNF-TrkB signaling cascade may be involved in the inflammation-induced heat hyperalgesia mediated by Cdk5 in the spinal cord in rats. Here we examine the effects of administration of the Cdk5 inhibitor roscovitine on heat hyperalgesia induced by CFA in regards to BDNF synthesis and transport, and TrkB protein expression.

## Materials and Methods

### Animals and experimental design

A total of 141 adult male Sprague-Dawley rats (200–250 g) were used in this study. All procedures were approved by the Committee of Animal Use for Research and Education of Shanghai JiaoTong University School of Medicine, and were in accordance with the guidelines established by the Ethical Issues of the International Association for the study of pain [18]. Rats were housed at a temperature of 22±4°C under a standard 12/12 h light/dark cycle and had free access to food and water. The animals were allowed to acclimate to the housing facilities for 1 week prior to beginning the experiments, and all efforts were made to minimize stress and the number of animals used.

### CFA administration

The first experiment was conducted to test the effects of CFA on heat hyperalgesia in rats. CFA (100 µl, Sigma, St. Louis, MO) was injected into the plantar surface of the left hind paw of six rats per group. As a control, saline (100 µl) was injected into the plantar surface of the left hind paw of rats per group. Behavioral analysis and cell testing were performed as described below.

### Catheter insertion and drug administration

Drugs were delivered by intrathecal injection as previously described, with slight modifications [19]. Briefly, rats were anesthetized with 4% pentobarbital (40 mg/kg) and a catheter (PE-10) was inserted at the lumbar level of the spinal cord between lumbar vertebrates 4 and 5 (L4 and L5). In our study, the skin was closed with two 4–0 nylon sutures, and the wound was covered with a mixture of polymixin B, neomycin, and bacitracin ointment once daily for 2 days. After recovery from anesthesia and surgery, verification of the proper placement of the catheter was confirmed by injecting 5 µl of 2% lidocaine flushed with 15 µl of saline. Following this procedure, successful insertion of the catheter was confirmed if the rats presented with impairment of motor function of their hind legs 10 s after lidocaine administration. After surgery (for five days), 5 µl of roscovitine (50 µg; 100 µg; Sigma, St. Louis, MO, R7772) or K252a (0.1 mg; 0.2 mg; Sigma, St. Louis, MO, K1639), dissolved in 10% DMSO dissolved in saline, TrkB-IgG dissolved in saline (15 µg and 30 µg Santa, sc-2027) was individually administered, followed by injection of 15 µl sterile saline 0.5 h before CFA treatment for one time in this experiment, respectively (n = 6/group). Unless otherwise specified, 100 µg roscovitine was administered in all experiments. The same concentration and volume of the vehicle were delivered in the same fashion and served as the vehicle control (n = 6/group).

### Behavioral test

Heat hyperalgesia was quantified by measuring the paw withdrawal latencies (PWLs) to radiant heat stimulation as previously described [20]. Briefly, radiant heat was directed to the plantar surface of each hind paw through a 1 mm thick glass plate. To avoid burning the skin of rats, the cut-off limit of exposure was 20 s. The withdrawal time between the onset of the stimulus and manifestation of the paw withdrawal response was recorded as the thermal nociceptive latency period. Prior to the behavioral test, rats were allowed to acclimate to the chamber for 30 min, and then the thermal stimulus was delivered to the ipsilateral and contralateral hind paws of rats in all groups. PWLs were measured at 0, 6, 24 and 72 h post CFA in the inflammation group and saline adminstration in the control group as an average of three trials for each hind paw. The interval of PWL measurement between ipsilateral and contralateral paws was five minutes. Measurement of PWL of rats in the vehicle and roscovitine or K252a pretreatment groups was taken in a similar fashion.

### BDNF enzyme-linked immunosorbant assay

Rats from the respective treatment groups were euthanized by overdose with pentobarbital (>100 mg/kg i.p). The spinal cord dorsal horn tissues of L4–L5 from the left side were removed and immediately homogenized after being weighed (50 mM Tris, pH 7.4, 150 mM NaCL, 1.5 mM MgCL2, 10% glycerol, 1% Trition X-100, 5 mm EGTA, 0.5 ug/ml leupetin, 1 mM PMSF, 1 mM Na3VO4, 10 mM NAF, and proteinase inhibitor cocktail) The BDNF levels (pg/mg protein) were determined by using the Chemikine BDNF kits (Chemicon,CYT306) following the protocol of the manufacturer.

### Protein extraction, immunoprecipitation, and Western blot analysis

Tissues of the spinal cord dorsal horn of the L4/L5 left sides of the spinal segment from treatment groups were removed and immediately homogenized in ice-cold lysis buffer (50 mM Tris, pH 7.4, 150 mM NaCL, 1.5 mM MgCL_2_, 10% glycerol, 1% Trition X-100, 5 mM EGTA, 0.5 µg/ml leupetin, 1 mM PMSF, 1 mM Na_3_VO_4_, 10 mM NAF, and a proteinase inhibitor cocktail). Homogenates were centrifuged at 12,000× g for 15 min at 4°C and protein concentrations determined using the BCA assay kit (Pierce, Rockland, IL).

Protein samples (30 µg) were separated by SDS-PAGE (sodium dodecyl sulfate polyacrylamide gel electrophoresis) and transferred onto PVDF membranes. After blocking with 1% bovine serum albumin (BSA) in TBST (50 mM Tris-HCL, pH 7.5, 150 mM NaCl, 0.05% Tween 20) for 1 h at room temperature, membranes were incubated overnight at 4°C with the appropriate primary antibody (anti-Cdk5, 1∶200, Abcam [Cambridge, MA] ab115812; anti-TrkB, 1∶500, Abcam [Cambridge, MA] ab18987). Membranes were washed in TBST between all incubations. Blots were then incubated with a 1∶1000 dilution of horseradish peroxidase (HRP)-conjugated secondary antibody (goat anti-rabbit or -mouse) for 1.5 h at room temperature and bands visualized with an enhanced chemiluminescence kit (Boehringer Mannheim, Indianapolis, IN) and exposure to x-ray files that were digitally analyzed using NIH Image software, version 1.60. The densities of specific bands were measured and normalized with an internal loading control band.

For immunoprecipitation, 50 µg of protein lysates from treatment groups of the L4/L5 left sides were incubated with 2 µg of Cdk5 antibodies or with IgG as a control at 4°C for 1 h. 40 µl of protein G Sepharose beads (Amersham Biosciences) pre-washed with 1× PBS was added and mixed at 4°C for 1 h. After washing with lysis buffer six times, the immunoprecipitated protein and its associated proteins were analyzed by SDS-PAGE and Western blot analysis.

### Statistical analysis

All data were presented as mean ± standard error of the mean (SEM). SAS 8.01 (version 8.01, SAS Institute Inc., Cary, North Carolina, USA) for Windows was used for statistical analysis. The time course of the effects was analyzed by a two-way analysis of variance (ANOVA) followed by a Dunnett's test. A one-way ANOVA followed by the Student Newman–Keuls' test was used to analyze the expression of BDNF and TrkB. A value of p<0.05 was considered statistically significant.

## Results

### 1. Heat hyperalgesia induced by intraplantar CFA injection was significantly reversed by intrathecal administration of roscovitine or K252a and TrkB-IgG

**Figure 1:**
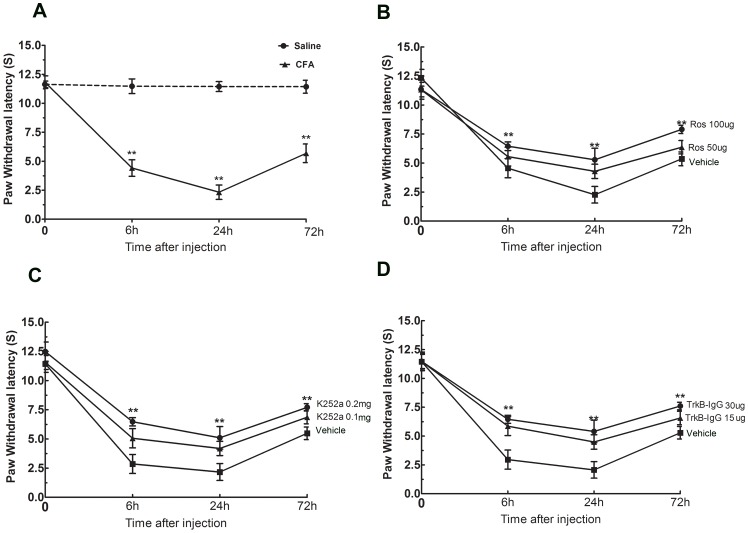
Heat hyperalgesia induced by CFA peripheral injection was significantly inhibited by intrathecal administration of roscovitine, K252a and TrkB-IgG. PWL to thermal stimuli significantly decreased following intraplantar injection of CFA,**P<0.01, (n = 6/group) (A). PWL to thermal stimuli significantly increased following intrathecal injection of roscovitine 0.5 h prior to CFA injection compared to the PWL of the control group. **P<0.01, (n = 6 per group) (B). PWL to thermal stimuli significantly increased following intrathecal injection of K252a 0.5 h prior to CFA injection compared to the PWL of the control group. **P<0.01, (n = 6, per group) (C). PWL to thermal stimuli significantly increased following intrathecal injection of TrkB-IgG 0.5 h prior to CFA injection compared to the PWL of the control group, **P<0.01, (n = 6, per group) (D).

**Figure 2:**
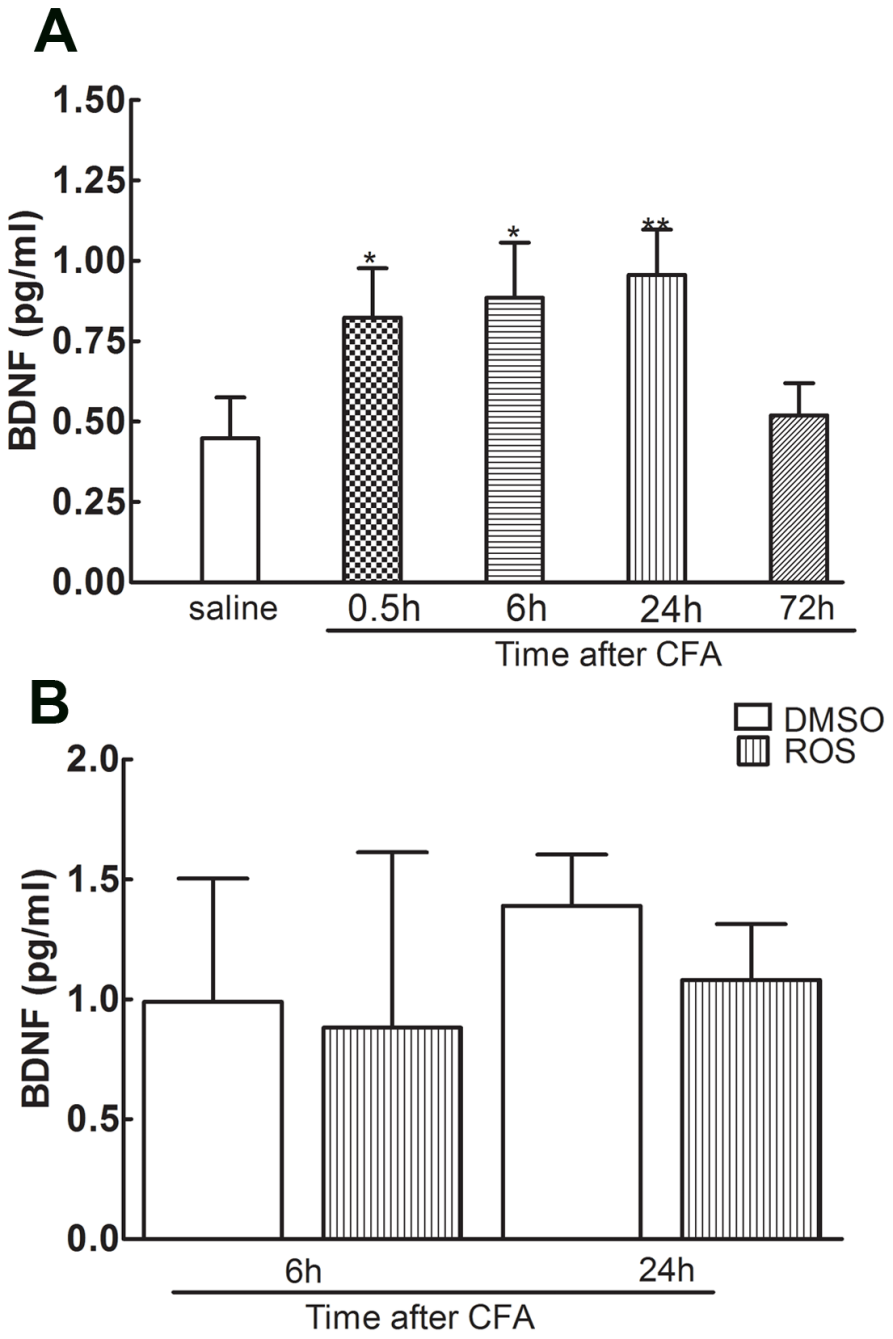
BDNF release was significantly increased in the spinal cord dorsal horn following intraplantar injection of CFA, and intrathecal adminstration of roscovitine had no effects on BDNF release. BDNF release significantly increased between 0.5*P<0.05, **P<0.01. (n = 6, per group) (A). No significant difference was observed between the roscovitine and control groups 6 hours and 24 hours after CFA injection, *P*>0.05. (n = 6, per group) (B).

Following injection of CFA into the left hind paws of rats, the maximal heat hyperalgesia occured 6 h after CFA injection and persisted for 24 h in our model. The average thermal PWL threshold of the ipsilateral hind paws was significantly reduced after CFA injection from 6 h to 72 h compared to the left hind paws of the control group (Figure 1A, **P<0.01). Furthermore, the average ipsilateral paw PWL thresholds were remarkably increased with treatment of 50 µg or 100 µg roscovitine 30 min before intraplantar CFA injection in a dose-dependent manner between 6 h and 72 h after CFA injection compared to the control group (Figure 2C, **P<0.01). In addition, the average left paw PWL thresholds were markedly increased with treatment of 0.1 mg or 0.2 mg K252a 30 min before intraplantar CFA injection in a dose-dependent method between 6 h and 72 h after CFA injection compared to the control group (Figure 2B, **P<0.01). The average left paw PWL thresholds were also clearly increased after treatment with TrkB-IgG compared to the control (Figure 2D, **P<0.01).

### 2. Intrathecal adminstration of roscovitine has no obvious effects on increased BDNF release from CFA injection

Previous studies demonstrated that the amount of BDNF release was significantly increased because of inflammatory stimulus. We examined BDNF release and the effects of spinal adminstration of roscovitine on BDNF release in the dorsal horn neurons of L4/L5 left spinal cord by ELISA after CFA injection (Figure 2A). BDNF release was significantly increased between 0.5 h and 24 h after CFA injection compared with the control group (Figure 2A, *P<0.05, **P<0.01). However, there was no significant difference between the roscovitine group and control group 6 h and 24 h after CFA injection (Figure 2B, *P*>0.05), suggesting that intrathecal administration of roscovitine did not have obvious effects on the release of BDNF.

### 3. Increased TrkB protein level caused by CFA challenge was reduced by pretreatment with roscovitine

**Figure 3:**
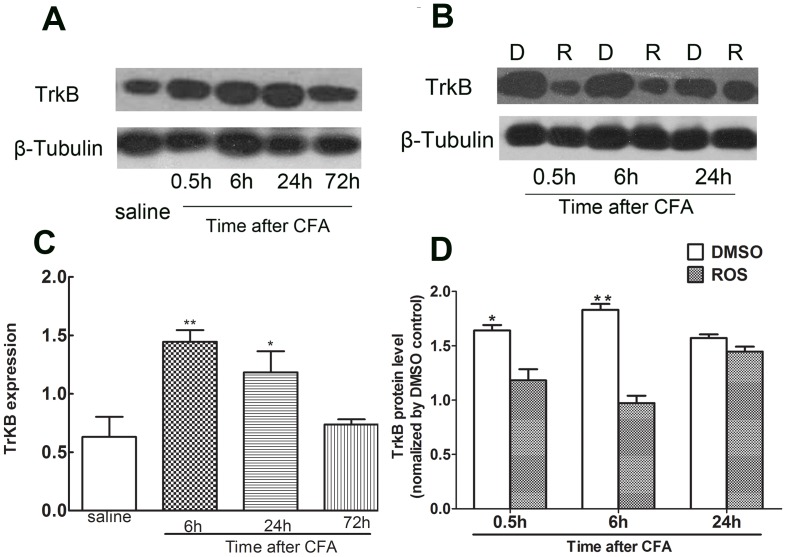
TrkB protein levels were significantly increased in spinal cord dorsal horns and reduced by roscovitine. Compared to the control group, TrkB expression was significantly increased from 0.5*P<0.05, ** *P*<0.01. (n = 5, per group) (A). Increased TrkB levels were significantly decreased by intrathecal injection of roscovitine (R) compared to control group (D) between 0.5 h and 6 h. *P<0.05, ** *P*<0.01(n = 5, per group) (B).

Previous studies demonstrated that the maximum heat hyperalgesia induced by CFA injection occurred at 6 h and lasted at least 24 h after CFA injection [21]. Therefore, we next analyzed TrkB protein in L4/L5 left spinal horn cords by Western blot after CFA injection. Results in left spinal horn cords demonstrated that TrkB was significantly upregulated post-intraplantar injection of CFA and decreased by 72 h compared to the control group (Figure 3A, *P<0.05, ** *P*<0.01). Furthermore, the increased levels of TrkB were significantly decreased by administration of roscovitine between 0.5 h and 6 h in the vehicle group vs. in Ros group, (Figure 3B, *P<0.05, **P<0.01).

### 4. Enhanced interaction of TrkB with Cdk5 following CFA challenge

**Figure 4:**
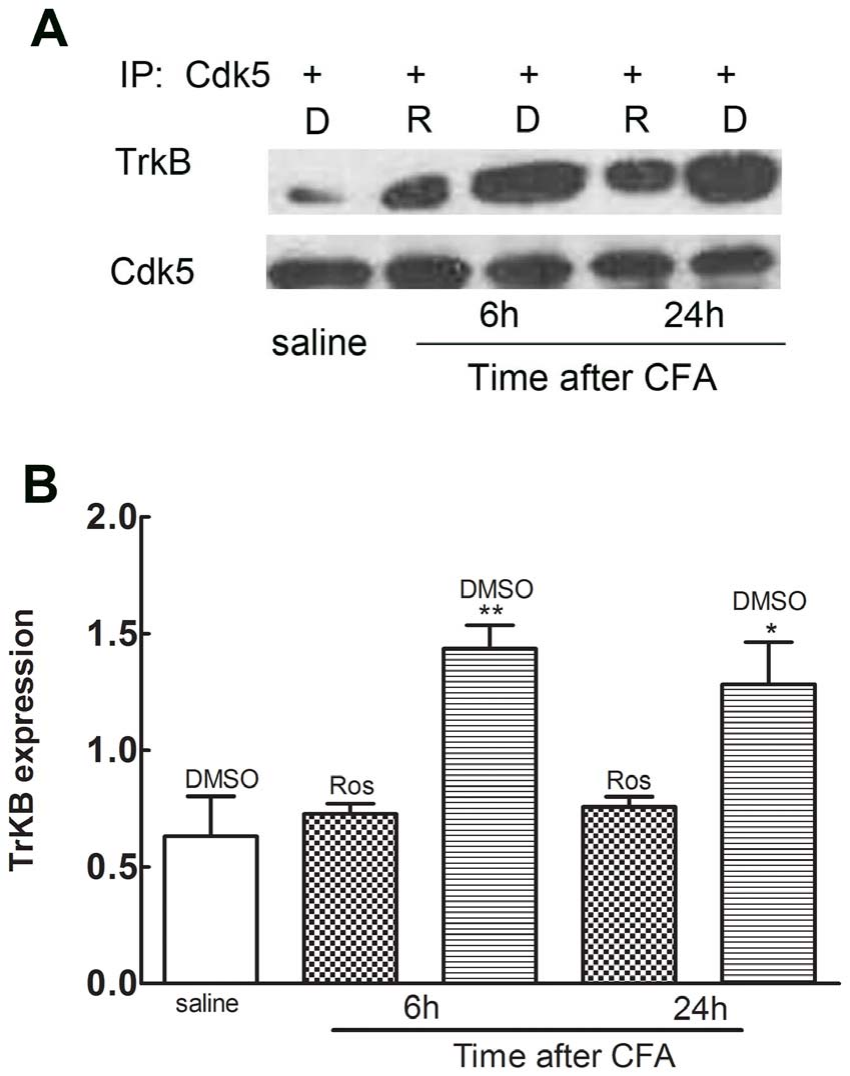
TrkB binding to Cdk5 in spinal cord dorsal horns was enhanced peripheral injection of CFA. Co-immunoprecipitation of TrkB with Cdk5 was significantly increased after CFA injection as detected by Western Blot analysis. TrkB binding was significantly decreased upon intrathecal injection of roscovitine 6 hours and 24 hours after intraplantar injection of CFA. *P<0.05, (n = 5, per group).

Although the upregulation of TrkB after CFA infection was decreased by administration of roscovitine, this data was not sufficient to confirm the close and direct interaction between Cdk5 and TrkB. Therefore, we performed co-immunoprecipitation assays to assess Cdk5 and TrkB binding in L4/L5 left spinal horn cords 6 h after intraplantar injection of CFA. Compared to the control group, the amount of TrkB that co-precipitated with Cdk5 was significantly increased after CFA injection (Figure 4, *P<0.05). Compared to the vehicle group, the enhanced TrkB binding to Cdk5 was decreased by roscovitine (Figure 4, *P<0.05).

## Discussion

The present study demonstrated the involvement of the BDNF-TrkB signaling pathway in CFA-induced heat hyperalgesia mediated by Cdk5. Previous studies had shown that the maximum heat hyperalgesia induced by CFA injection began 6 h after injection and lasted at least for 24 h [21]. In agreement with previous reports, in our study, the peak levels of heat hyperalgesia occurred from 6 h to 24 h after CFA injection. Moreover, the amount of BDNF release was significantly increased between 0.5 h and 24 h, and returned to baseline levels 72 h after CFA injection.

The results from previous studies indicated that the increased BDNF release had activated TrkB during inflammatory pain [22]. Data presented in our study showed that TrkB protein level was remarkably increased between 0.5 h and 24 h after CFA injection, and heat hyperalgesia was inhibited by intrathecal injection of K252a following spinal intrathecal injection of K252a 0.5 h after peripheral injection of CFA. Although K252a is an inhibitor of TrkA, TrkB and TrkC, K252a mainly blocks BDNF binding to TrkB in spinal cord level in our model, based on the following evidence: 1) TrkB has been shown to be a high affinity receptor of BDNF over TrkA and TrkC [23]; and 2) intrathecal administration of the BDNF-scavenging protein TrkB-IgG resulted in the increased threshold of thermal hyperalgesia induced by inflammation [9]. Four neurotrophin family members bind the Trk receptors with high affinity, including nerve growth factor (NGF) that binds TrkA, BDNF and neurotrophin-4 (NT-4) that binds TrkB neurotrophin-3 (NT-3) that binds TrkC. However, NT-3 and NT-4 seem to have no significant pronociceptive role in the spinal cord dorsal horn during chronic pain [24]. NGF acts mainly as a peripheral mediator during inflammatory pain, while BDNF acts mainly as a central modulator of inflammation-induced hyperalgesia [25]. On the other hand, in vitro studies had shown that Cdk5/p35 knockdown or administration of K252a resulted in a significant decrease of TrkB expression [16], [17]. Nevertheless, to further identify the effects of BDNF on TrkB in our model, the BDNF-TrkB-specific scavenger TrkB-IgG was delivered from day 1 to day 5 after CFA. The results demonstrated that the heat hyperalgesia induced by CFA was markedly inhibited by TrkB-IgG and that the BDNF-TrkB signaling pathway played a key role in the heat hyperalgesia induced by CFA. However, TrkB-IgG is only a scavenger of BDNF and not a specific antagonist of TrkB. Therefore, to exclude the effects of K252a on TrkA and TrkC, we will need to design experiments in the future that assess TrkB expression inhibition using gene-based methods.

Previous studies reported that heat hyperalgesia was significantly reversed by intrathecal injection of 100 µg roscovitine without affecting the movement of rats in a CFA-induced inflammatory pain model [8]. However, the exact mechanism by which Cdk5 mediates heat hyperalgesia induced by inflammation still remains unknown. In the CA1 region of the hippocampus in mice, Cdk5 or p35 knockdown led to a significant decrease of TrkB [16], [17]. In addition, considerable evidence demonstrated that the BDNF-TrkB signaling pathway is involved in mediating the pain sensitization of inflammatory pain [18]–[20], [26]–[28]. BDNF is the most abundant and widely distributed neurotrophin in the central nervous system (CNS). In primary sensory neurons, BDNF is prominently distributed in small or medium diameter neurons of DRG and is anterogradely transported from the cell bodies of these cells to terminals in the spinal cord dorsal horn.

In our study, we measured the BDNF level in the spinal cord dorsal horn by ELISA. Our results showed that BDNF level was significantly increased between 0.5 h and 24 h post CFA treatment. Given that Cdk5 serves a major role in controlling the release of neurotransmitters [29], [30] together with our previous studies demonstrating that synaptophysin, a membrane protein that functions in the endocytosis-exocytosis cycle of synaptic vesicles to transport neurotransmitters, is involved in the inflammatory pain mediated by Cdk5 as well as the roscovitine-induced inhibition of the up-regulation induced by CFA [7], we sought to determine whether Cdk5 mediates heat hyperalgesia induced by peripheral injection of CFA by controlling the amount of BDNF. However, no substantial change in level of BDNF was observed between the roscovitine and control groups 6 h after CFA injection in the spinal cord, suggesting that spinal administration of roscovitine has no significant effects on BDNF release. One reason for this may be that synaptophysin mainly functions in transporting the small synaptic vesicles [31]. However, BDNF is prominently stored in large synaptic vesicles [32], [33]. A recent report showed that the increased inhibitory postsynaptic currents (IPSCs) induced by BDNF were blocked by k252a or roscovitine in cerebellar Purkinje neurons [34]. In that study, roscovitine block TrkB from producing IPSCs as an inhibitor between BDNF and TrkB, with no obvious effects on adjusting the amount of BDNF to mediate the IPSCs. The study also demonstrated that Cdk5 acted on TrkB rather than on BDNF. Their results of experiments perhaps help us explain the reason for why in our study, roscovitine had obvious effects on TrkB expression, but not on the release of BDNF. However, it may be the difference between the endogenous and exogenous BDNF to roscovitine. In addition, another study revealed that BDNF induces p35 expression and Cdk5 activity in PC12 cells, suggesting that BDNF regulates the activity of Cdk5 as an upstream regulator [35]. Nevertheless, future experiments will be designed to analyze the relationship between Cdk5 and BDNF in our model.

Considerable studies have demonstrated that TrkB plays a decisive role in mediating heat hyperalgesia induced by inflammation [36], [37], and TrkB is significantly upregulated by chemical, thermal, inflammatory mediators or mechanical noxious stimulation in the spinal dorsal horn. In our study, TrkB was markedly upregulated between 0.5 h and 24 h after peripheral injection of CFA. Previous studies demonstrated that Cdk5 or p35 knockdown led to significant decrease of TrkB expression in primary hippocampal neurons, thus we wondered whether spinal administration of roscovitine had significant effects on TrkB protein expression in our study. As predicted, increased TrkB protein induced by peripheral injection of CFA was markedly decreased by roscovitine between 0.5 h and 6 h after CFA injection. In addition, the heat hyperalgesia induced by peripheral injection of CFA was significantly inhibited by administration of roscovitine or K252a from 6 h to 72 h, respectively. Taken together, it seems likely that the pathway of BDNF binding to TrkB may be blocked by roscovitine. Therefore, immunoprecipitation was used to test the interaction between Cdk5 and TrkB in the spinal cord dorsal horns of CFA-treated rats. Our results showed enhanced TrkB binding to Cdk5 6 h and 24 h after intraplantar injection of CFA, and the increased TrkB binding was significantly decreased by roscovitine. Although Cdc2, Cdk2, Cdk5, and Erk1/2 kinase activity can be significantly inhibited by roscovitine [38], our previous paper had accounted for the reasons why roscovitine mainly inhibits Cdk5 activity rather than other Cdks in adult rats [7]. Therefore, spinal adminstration of roscovitine mainly reduced the increased TrkB by inhibiting Cdk5 activity. Thus, it is likely that roscovitine reversed the heat hyperalgesia induced by peripheral injection of CFA by blocking the linkage between BDNF and the TrkB signaling pathway in rats.

## Conclusion

The current study demonstrated that roscovitine reversed the heat hyperalgesia induced by CFA via blocking the BDNF/TrkB signaling pathway. Spinal administration of roscovitine or K252a significantly alleviated the CFA-induced heat hyperalgesia. Increased TrkB protein levels were significantly reversed by roscovitine, which may be of great importance in blocking the interaction between Cdk5 and TrkB, inducing and developing the early stage of chronic inflammatory pain. Consequently, we hypothesize that interrupting the linkage between Cdk5 and the BDNF/TrkB signaling pathway may present a therapeutic target for the treatment of inflammatory pain.
